# Exploring the challenges of virtual internships during the COVID-19 pandemic and their potential influence on the professional identity of health professions students: A view from Qatar University

**DOI:** 10.3389/fmed.2023.1107693

**Published:** 2023-01-30

**Authors:** Hiba Bawadi, Rula Shami, Alla El-Awaisi, Ayad Al-Moslih, Hanan Abdul Rahim, Xiangyun Du, Joyce Moawad, Ghadir Fakhri Al-Jayyousi

**Affiliations:** ^1^Department of Human Nutrition, College of Health Sciences, QU Health, Qatar University, Doha, Qatar; ^2^Department of Public Health, College of Health Sciences, QU Health, Qatar University, Doha, Qatar; ^3^College of Pharmacy, QU Health, Qatar University, Doha, Qatar; ^4^College of Medicine, QU Health, Qatar University, Doha, Qatar; ^5^College of Education, Qatar University, Doha, Qatar

**Keywords:** virtual training, professional identity, university students, COVID-19, Qatar, health professions

## Abstract

**Introduction:**

COVID-19 has imposed many shared limitations on medical and health education. Just like other health professions programs at most institutions, the Qatar University health cluster (QU Health) applied a containment approach and shifted all learning online, and onsite training was replaced by virtual internships (VIs) during the first wave of the pandemic. Our study aims to explore the challenges of virtual internships during the COVID-19 pandemic and their influence on the professional identity (PI) of the health cluster students from the College of Medicine, the College of Health Sciences, and the College of Pharmacy at Qatar University.

**Methods:**

A qualitative approach was employed. In total, eight focus groups with students (*N* = 43) and 14 semi-structured interviews with clinical instructors from all the health cluster colleges were conducted. Transcripts were analyzed following the inductive approach.

**Results:**

The major challenges reported by students were mainly related to the lack of the required skills for navigating the VI, professional and social stressors, the nature of VIs and the quality of learning, technical and environmental issues, and the development of students' professional identity in an alternative internship environment. The challenges relating to the development of professional identity included: limited clinical (practical) experience, a lack of experience in fighting a pandemic, a lack of communication and feedback, and a lack of confidence in meeting the internship's goals. A model was constructed to represent these findings.

**Discussion:**

The findings are important in identifying the inevitable barriers to virtual learning for health professions students and provide a better understanding of how such challenges and different experiences would be affecting the development of their PI. Hence, students, instructors, and policymakers alike should strive to minimize these barriers. Since physical interactions and patient contact are indispensable components of clinical teaching, these extraordinary times demand innovations involving technology and simulation-based teaching. There is a need for more studies that are focused on determining and measuring the short- and long-term effects of the VI on students' PI development.

## 1. Introduction

COVID-19 has imposed many shared limitations on medical and health education. Just like other health education programs at most institutions, the Qatar University health cluster (QU Health), which constitutes the group of Colleges of Medicine, Health Sciences, Pharmacy, and Dental Medicine at Qatar University, applied a containment approach and shifted all learning online, and onsite training was replaced by virtual internships (VI). Students from all clinical programs were removed from practice sites to reduce the risk of transmission and allow these sites to focus on essential services.

Early clinical training, which applies practice-based-learning models, is the backbone of medical education (1) and plays a pivotal role in enhancing clinical skills development across all medical professions (2, 3). There is empirical evidence that early experience in medical education strengthens medical students' learning and makes it more real and relevant (4). Several competencies that healthcare students need to build during their training and that supplement their clinical knowledge and skills have been defined in literature (4–12). Examples include interpersonal competencies (communication and collaboration), cognitive skills (problem-solving, critical thinking, and reflectivity), work-related skills (planning and time management), and professionalism (integrity, sense of responsibility, respect, and empathy). It is therefore important to facilitate and assess these competencies in the context of the complexities of real-life situations (12). Moreover, a set of important outcomes of early clinical learning include student satisfaction, adapting to clinical environments, reducing stress and enhancing confidence upon interacting with patients, and learning self-reflection and appraisal skills.

Another important outcome of early clinical learning is professional identity (PI) formation (4–10, 12, 13), where PI is defined as “the attitudes, values, knowledge, beliefs and skills shared with others within a professional group” (4). A simple model by Vivekananda-Schmidt et al. shows that medical students' perceived key factors for building their PI were their extent of participation, acknowledgment of their professional roles, and their independent practice of professional activities (14). Literature has consistently shown how PI formation is socially constructed (5, 6, 11), which allocates great importance to communities of practice-based and situated learning that incorporates experiential and reflective learning processes, formative feedback, use of personal narratives, availability of a role model, and candid discussions (5, 15). Practical competence and PI are, therefore, interlinked concepts and constitute the two important qualities that empower medical students to play an indispensable role in helping patients (3, 14, 16). Nevertheless, literature has reported several challenges to PI formation, which may be associated with the nature of healthcare settings and the extent to which these settings enable the building of essential health professional competencies (17, 18). The concept of professionalism, as one of the essential competencies, is also tightly contextual and influenced by culture (12, 19). For example, the Arab region, including Qatar, has reported a culture-related gap in some professional competencies, like the ability to learn independently, think critically, and practice reflection (17, 18).

Additionally, while evidence suggests that virtual teaching is effective (20, 21), it has been shown to present several barriers and challenges (22, 23), such as environmental challenges, a lack of technological resources and skills, and insufficient institutional support (24, 25). Institutions also continuously try to develop resources to improve student engagement and interactivity during online classes (26).

Although existing literature has extensively described the best practices for online medical education (20–23), further research is needed to understand how to apply such practices given the short time frame available when an unexpected situation arises, like a pandemic. Recent literature describes how being offsite and the lack of in-person interaction with patients and other healthcare professionals in the era of COVID-19 might add to the already existing challenges to online medical education and may prevent medical education from reaching its goals (1, 8, 26–30).

### 1.1. Virtual internships at QU health during the COVID-19 pandemic

During the COVID-19 pandemic, like many health professions programs around the world, QU Health was faced with the need to create safe and appropriate alternatives for onsite clinical training. Thus, onsite training was replaced by virtual internships (VI), which is practice-based learning *via* different online platforms, during the first wave of the pandemic (31).

The clerkships at the College of Medicine (CMED) were shifted to online platforms with an emphasis on the delivery of clinical knowledge and reasoning through means of case discussions. Such online sessions were delivered *via* platforms supported by QU, like Cisco Webex and Microsoft Teams. The MD program at CMED divided the time of the clerkship between online lectures and study sessions, and it replaced clinical placements until after the restrictions of clinical attachment were lifted in July 2020. Meanwhile, the program ensured that students were educated about the required clinical knowledge for their clinical placements. This time was also used for the completion of in-program assessments, such as case reports, case logs, case presentations, and work-based assessments. The assessment involved case discussion assessment. Students were asked questions about the history and physical examination findings of certain medical conditions. Questions covered medical conditions that patients would commonly present with. An assessment blueprint ensured fair distribution of questions across the curriculum content. Assessment also targeted clinical reasoning, diagnosis, investigation, and treatment across a range of clinical problems. Case discussions were conducted online, but subsequent case discussions were run in multiple mini-interview formats from home.

During the COVID-19 pandemic, the College of Pharmacy (CPH) clinical training also moved from experiential learning in the training sites to virtual internships (VI). The full-time PharmD program includes eight advanced pharmacy practice internships, each lasting 4 weeks, based on situational learning through internships. Students are continuously assessed throughout the program, through mid- and final-point evaluations, according to 23 predetermined criteria mapped to seven educational outcomes, which are assessed by their assigned preceptor for each internship. Rotation activities include journal clubs, case presentations, and therapeutic discussion topics. Students must then complete one final exit examination known as an oral comprehensive examination, all of which were conducted virtually during the spring 2020 semester.

The Department of Public Health at the College of Health Sciences shifted its health education practicum to a project-based internship (PBI), replacing their clinical training in Primary Healthcare Centers (32). In the PBI, students constructed questionnaires after reviewing the literature to assess the needs of specific audiences regarding different public health topics. They collected online data and conducted the analysis. Then, they delivered online activities to promote the health of the chosen audiences. Students also built creative models to plan for implementing these activities and services and suggested a variety of methods to monitor the implementation process. In addition, they planned for effect evaluation by suggesting specific outcome indicators and different research designs.

The Human Nutrition internship at the College of Health Sciences is a structured program that includes rotations of clinical sites, such as cardiac, oncology, renal, food service, and other medical departments of Hamad Medical Corporation (HMC) hospitals (33). During the pandemic, most clinical rotations were immediately shifted to virtual rotations, and various student tasks were suspended, such as patient education, counseling, and working in interdisciplinary teams. These were replaced by an extended number of hours of professional development classes, where students attended workshops led by dietitians abroad and got more involved in case simulations using the telehealth concept, case studies, case presentations, and preparation of awareness campaigns and by writing articles.

Qatar has invested heavily in improving the health and education sectors recently and placed them in the top priorities of the country's vision. Given the uncertainty of the future, should the COVID-19 pandemic persist, there is a need to identify the challenges faced by university students in Qatar, across different healthcare disciplines, due to the shift to virtual internship. Understanding the students' perspectives, as well as those of faculty members, would be a way to improve their experiences with virtual training and to make sure this training continues to serve its role in shaping their identity as future health professionals. Therefore, the purpose of this study is to explore the challenges posed to students by the shift to VI in the era of COVID-19 and the potential threats to students' professional identity from the perspective of QU Health students and clinical faculty. This could be a major step in guiding plans aimed at maintaining the optimal standards of health professions education in Qatar during emergencies and beyond.

## 2. Methods

### 2.1. Study design

An inductive qualitative approach was used to capture and describe the perspectives of QU Health students and their clinical faculty members. With this approach, the researcher is not biased or occupied with predetermined assumptions and is open to understanding the phenomenon and answering the research question (34). Focus group discussion was chosen for students and was conducted in parallel with semi-structured interviews for clinical faculty members. The research team, consisting of faculty members from QU Health and College of Education, reviewed the relevant literature, discussed the findings, and then developed an interview guide and a focus group guide (see Supplementary Appendices 1, 2). The study was conducted around the end of the spring 2020 semester, during the first wave of the COVID-19 pandemic.

### 2.2. Sample population

Purposive sampling was conducted to recruit participants who were considered rich cases and would help us understand the challenges that the VI poses to students. The study included a total of 43 students from QU Health who registered for their clinical training. Twelve students from the PharmD program at the College of Pharmacy (CPH), 11 students from the Public Health (PH) program, 9 from the Human Nutrition (HN) program, and 11 from the College of Medicine (CMED) were invited to participate and all accepted the invite. Fourteen clinical faculty members who supervised a VI during the pandemic individually or alongside a preceptor from the clinical site were also invited.

### 2.3. Data collection

Emails were sent out to all QU Health students who were undertaking clinical internships and to clinical faculty members. The email included a description of the study and an invitation to participate through an online platform. The invitation included a consent form, which participants were asked to sign electronically or to sign and scan when convenient before joining the focus groups or interviews. This was followed by two reminder emails.

A date and time were communicated, along with a calendar invitation and a link to the WebEx online platform. At the beginning of the focus group discussion and the interviews, the facilitator (a trained member of the research team who is not involved in delivering the VI) reiterated the contents of the consent form, explained the rules of conduct in the group, and informed them that the sessions would be recorded. The focus groups and interviews were conducted in English, each lasting 60–70 mins and 45 mins, respectively. Data saturation was reached where no new information was reported by students and faculty members after conducting these discussions and interviews.

### 2.4. Data analysis

The focus group discussions and interviews were recorded *via* WebEx. Generated transcripts were validated as verbatim and used for analysis by a research team member who was not involved in the focus group discussion nor in conducting the individual interviews. Inductive qualitative analysis was employed to identify the themes and subthemes related to the challenges of VIs in the focus group discussions and individual interviews. Initial coding was conducted to identify these themes and a codebook was constructed. Coding is defined as the process of labeling, organizing, and structuring qualitative data to find themes and patterns (35). Each transcript was coded and new themes were added to the codebook as they emerged. Constant comparisons were conducted to differentiate one theme from another. With each addition of new data, themes were added and modified as needed. Finally, the themes were combined into a coherent, textural description of the phenomenon. To assist in the verification process, three members of the research team analyzed the data independently by reading through the transcripts and identifying the common themes separately. They then came together to discuss the results and reach a consensus regarding the themes and categories. First, the focus group discussions were analyzed, followed by the individual interviews.

## 3. Results

**Figure 1:**
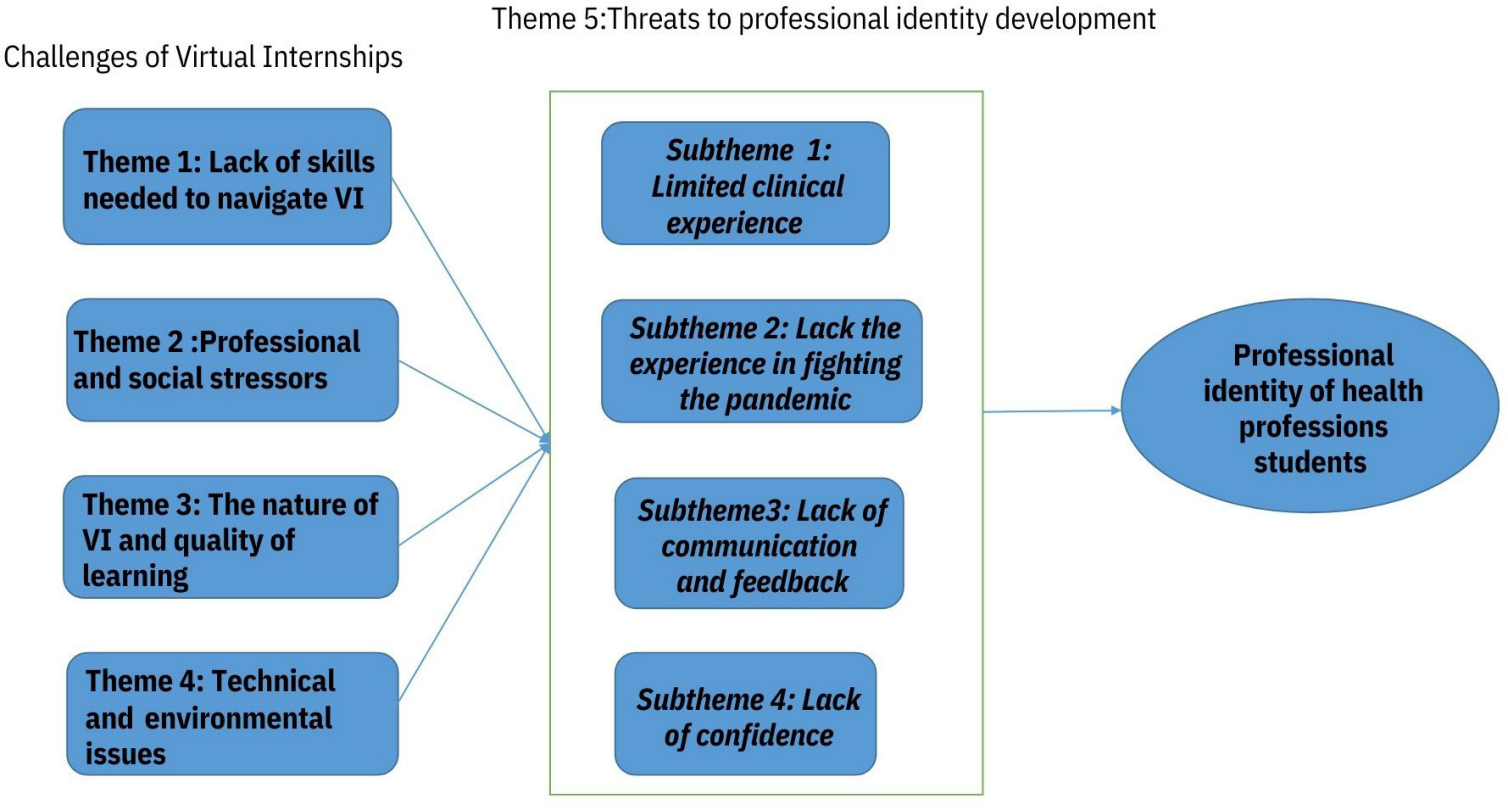
The themes and sub-themes emerged from the data and their relationship with professional identity.

In total, we conducted eight focus groups with students (*N* = 43) and 14 interviews with clinical instructors from all the QU Health colleges. The major challenges reported by students were mainly related to a lack of the skills needed to navigate the VI, professional and social stressors, the nature of VIs and quality of learning, technical and environmental issues, and the development of students' professional identity in an alternative internship environment. The themes and sub-themes emerged from the data, and their relationships with professional identity are presented in Figure 1.

### 3.1. Theme 1: Challenges related to a lack of the skills needed to navigate the VI

Faculty members expressed that a few students had insufficient communication and information-searching skills and needed to develop their online skills. The Public Health (PH) training coordinator mentioned that students were challenged in navigating the online skills, setting up their presentations, using the chat box, and participating in the discussions. One student explained:

“Maybe because our topic was about COVID-19. The challenge was it is a new, emerging disease that is happening now, so the process of collecting information and also the interventions themselves” (sic) (PH Student).

Both students and faculty members of CMED perceived a need for the development of new technological skills, like reading a detailed CT scan. This was specifically applicable to some older faculty members.

“How to open the back system, that's the X-ray system, how you show CT scan live on the WebEx that needs some help of the IT [information technology]. You can show a slice of CT scan on a little presentation… that's not the problem. But if you want to scroll a CT scan going from one pixel to another… that need a bit of IT help” (sic) (CMED Faculty 4).

### 3.2. Theme 2: Challenges related to professional and social stressors

To the majority of students, the VI imposed a feeling of stress and uncertainty in many ways. A student from HN expressed this challenge, saying, “I was stressed and not able to work on my training assignments and tasks as how I used to do in my usual routine before the crises” [sic]. According to a CMED faculty member, “The fear of getting and of completing on time, getting promoted to the next year, getting graduated on the expected year” [sic] were also among the main concerns that students had. Students' stress about the situation was aggravated by insufficient communication from the university regarding future plans for their training. This created a sense of frustration and uncertainty about the future regarding what they would be doing next.

“I'm worried that, if this situation (COVID-19) continues, that we will still be lacking enough experiences on counseling patient or that some policies or guidelines changes that we may not have time to adjust to or having experience on it” (sic) (HN Student 4).

“We need more communication and things need to be, at least to students, I mean, assessment, curriculum, and resources, because now we are in this situation, it is not a normal thing. So, relieving the stress of the situation can be with better communication… So the college need to decide, take some decisions, and unusual decisions and for us; everything need to be clear, the future plan, curriculum, resources and assessment” (sic) (CMED Student 9).

Students struggled with the feeling of being isolated while working from home and expressed being challenged by a lack of social interactions with their classmates and instructors, which had always been an important aspect of their on-campus life.

“When presenting online, they [students] missed social interaction. In terms of content, everything was there, no problem. They missed the social interaction with their classmates, whom they are presenting for them” (sic) (PH Faculty 2).

“It is also the culture on campus. Our students would like to, they like to meet professors, talk to them, share their concerns, hear a feedback, address their needs, and we have been good listeners to them. So, that was a big switch, big change once we were unable to meet those students as before. Sometimes, they stopped by the office three times a week and send tons of emails in normal situation. And in a sudden, we were unable to have those students in the office” (sic) (PH Faculty 1).

Being in front of screens all day instead of being on training sites and in hospitals also made students feel demotivated and in need of support and mentorship.

“I'm struggling with motivation also, because when we used to go to the hospital, you feel motivated when you take care of the patient and you are a part of the team, whereas when you're home and you just have lecture” (sic) (CMED Student 1).

“We need motivation, guidance, and kind of follow-up, which is actually the role of mentors… So if we had, for example, mentors from the college, and every four or five students with one mentor, from the staff of the college, they ask about them, they follow them, they guide them, this would be much better” (sic) (CMED Student 9).

“I agree with the lack of motivation and I think the main reason for it personally, for me, is because during these online lectures, as much as the doctors are trying their best to give us the information that there's always a huge lack of interaction. And it's very hard to engage…” (sic) (CMED Student 3).

### 3.3. Theme 3: Challenges related to the nature of VIs and the quality of learning

Each student mentioned at least three to four examples of different issues that they thought could have adversely influenced the quality of their internship. Almost all students agreed that the online lectures were missing the interactive component and were not engaging.

“It's very hard to engage during those. It's like listening to a recording. It's not the same and it's very difficult to concentrate sometimes… we don't get as much as we're supposed to” (sic) (CMED Student 3).

In addition, students from HN and PH expressed being overloaded with assignments and thought the VI was not resourceful and offered little benefit compared to real training. Some students explained this challenge:

“The most challenge I have faced was the huge amount of assignments I had to do. It was very hard for me and my colleagues and it was time consuming with very little benefits comparing with real training” (sic) (HN student 3).

“Some don't actually attend because it's a waste of time for some people and I feel that we have lack of resources, so there's a lot of dependence on self-directed learning (SDL) in our part, like, “Oh, here's the blueprint, it's SDL, all of it by yourself” (sic) (CMED Student 8).

### 3.4. Theme 4: Challenges related to technical and environmental issues

All students faced time management difficulties due to the home-working environment. It was difficult to sustain working hours from home due to the nature of the home environment and the disruptions that it caused. “It was really hard to achieve eight working in a day because you know you are at home, you still need to like to see what your family you want” [sic], one CPH student explained. They also expressed that working from home makes you work constantly with no breaks and causes issues with time management.

“I have a hard time fixing my sleep schedule. It was easy to fix because we have to wake up early, and then we come back study, and then we're exhausted by the by ten o'clock or nine. But now, like, a hard time” (sic) (CMED Student 5).

“Time management is a very big challenge. Staying at home all day and having many responsibilities beside the training made me very disorganized” (sic) (HN Student 1).

A few students also faced some technical issues related to Wi-Fi and connectivity and found it hard to spend long hours in front of screens.

“The sound sometimes break and the Internet, and all of those distractions at the end of the lecture, we don't get as much as we're supposed to” (sic) (CMED Student 3).

“The only challenge that I faced was looking at the screen for long time, which gives me headache, meaning that I will have to step away for an hour or so, which could affect me negatively, especially if I was attending a lecture or a meeting with the preceptor” (sic) (HN student 4).

### 3.5. Theme 5: Potential influence on students' professional identity

The majority of students were concerned about the threats that the VI posed on the development of their professional identity. Students talked about the limited clinical practice, lack of experience in fighting a pandemic, lack of communication and feedback from preceptors, and lack of confidence in meeting the training goals.

#### 3.5.1. Sub-theme 1: Limited clinical (practical) experience

An important challenge reported by all students and faculty members was the limited clinical experience offered to students during the pandemic. This was partly due to the limited number of cases and the subsequent small number of clinical interventions imposed. Students described the quality of patient cases they were dealing with as basic. Thus, students were unable to respond to real patient cases and requests. One student, who was doing the internal medicine rotation, mentioned:

“Unfortunately, they were barely admitting patients due to COVID-19 and they were saving the beds or rooms for COVID-19, so the patient cases or the patient that we had, they were not very interesting, and we missed the part of the follow-up” (sic) (CPH Student 4).

As a result, students were also challenged by the lack of onsite interactions with the healthcare team and the lack of opportunity to build connections with clinicians as role models, as explained by the students and faculty members.

“Online didactic lectures, which, it doesn't be that sense of connections with clinicians. Unlike when they were taking them during the rotation, so having, you know, lack of encounter with the patients and at the same time lack of contact with clinical faculty and their expertise role modeling and all these things. I think that was also a challenge” (sic) (CMED Faculty 5).

“I think there's a lot of, I would say, you know, formal and formal verbal and non-verbal communication that can be accommodated, like, good habits or good, you know, traits. And then, I think that we need to try to avoid or manage, or even resist, in order to make sure that the communication is fruitful and actually, I'd say productive in the sense of educational process” (sic) (CMED Faculty 3).

Students were not able to explore the reality of the practice setting. Students argued that being away from practice sites prevented them from practicing and gaining clinical and non-clinical skills. Hence, students were challenged by their inability to understand the healthcare team perspective as well as the patient perspective when making correct recommendations.

“In the food service rotation, I see that we couldn't have the opportunity to see what is happening in the tray line. I was waiting for this rotation but couldn't have the ability to see what is happening there” (sic) (HN Student 3).

“A lot of the times, when virtually, when you see recommendation, you have no idea why the physician chose this. But in real discussions, when you're there with the physicians, and they're telling you what they see the patients, what they're expecting and other factors that aren't usually documented” (sic) (CPH Student 10).

Similarly, CMED students faced difficulty with conducting physical examinations of patients, taking patient history, and assessing physical findings.

“Students need to see and to face a patient also taking the history. You can't tell them, “Oh, this is possible asthma.” How we can ask about history of wheezes, family history, you know the long list of, and how you play with the history. But when they face a real patient, not every patient is the same” (sic) (CMED Faculty 4).

#### 3.5.2. Subtheme 2: Lack of experience in fighting a pandemic

When the training sites suspended training for the semester, the majority of students were disappointed and explained how gaining no experience regarding their role in fighting a pandemic would affect them as future clinical practitioners. They expressed that the VI allowed no time for them to gain such knowledge or experience, nor to help in the crisis.

“Hamad [hospital] is already swamped with patients, so finding the time and the resources to get this information or to be exposed to the information about how to deal with pandemics at this time point is not easy” (sic) (CPH Student 2).

“We signed up to be clinical practitioners regardless of what the situation is. So, I don't really see what's the difference of me or another pharmacist just being there in the front line, trying to help our patients” (sic) (CPH student 5).

Several CMED students tried to compensate by joining the fight as volunteers, and one of them expressed, “Some people say it's a risk for me as a student, and for my family, but at the end of the day, I don't like sitting down at home and not doing anything. So I prefer to volunteer during this pandemic and have an impact” (CMED Student 10).

The views of clinical faculty members widely coincided with those of students. They believed that students would be better off practicing in hospitals since this is their duty as doctors during a pandemic.

“… How to use The PPE [personal protection equipment], how to limit the chance of getting the infection once your doctor; you cannot say, “No sorry, I got kids.” Well, everybody got kids. It's our job. We are like soldiers, and you cannot say, “No I can't fight the enemy because I got kids” (CMED Faculty 4).

#### 3.5.3. Subtheme 3: Lack of communication and feedback

A number of students expressed being challenged by the level of communication and feedback from the faculty members and preceptors, who were busy with the pandemic and not able to provide students with the extent of support that was needed. Sometimes, technical issues hinder interaction with faculty members during online sessions.

“There was some technical difficulties, including sometime poor voice. You know, the slides share the slide. You cannot see the students if you have more than one student. The interaction being a big thing because you cannot answer all the questions the same time. You have to work a formula where you allow...” (sic) (CMED Faculty 5).

“Students have the right to ask questions, find their faculty, even if we have answers for extra time. Some of the skillset they need to experience with new patients, doing some of the images, like x-rays and CT scan, for example, we have to make sure that how a system that allows them to, even with the digital platform” (sic) (CMED Faculty 5).

The lack of face-to-face interactions posed a similar challenge to students regarding teamwork, collaboration, communication, coordination, and task distribution among their group members. One student explained, saying, “I have issues in teamwork. The communication and coordination was very difficult among us and you know online communication. Because it was online, it was a challenge to distribute the tasks among group members and review the work and what each student has to do” [sic].

The virtual nature of the internship also created another prominent challenge in regard to modes of assessment. It was difficult to assess clinical skills and the effectiveness and confidentiality of assessments suffered.

“From the technology point of view, the challenges of using remote assessment is that the same challenges will be the voice the and on the interaction… the students, if they know you by your name and they know your background, they always think that the question about your specialty” (sic) (CMED Faculty 5).

“And so, and then if we rely mostly on the OSCE [Objective Structures Clinical Examination] and MCQs [Multiple Choice Questions], and OSCEs is a major part of the assessment, and then how are we going to test the students on OSCE without having clinical replacement? So, this was the biggest challenge” (sic) (CMED Faculty 4).

#### 3.5.4. Subtheme 4: Lack of confidence

All the above-mentioned challenges created a feeling of low confidence that the VI, being not based on real-life experience, would meet its goals.

“For my side, I really wanted to wait and actually get an actual clinical rotation because for me, now, if I, let's say, got a job in Infectious Disease (ID), I have no idea what to do. I don't know how to approach the case, I don't know what's important, what's not important” (sic) (CPH Student 5).

“If this continues throughout the next all of the clerkship phase, then I wouldn't be very comfortable or confident enough to then be put in the hospital as soon as possible; we need to be put into the hospitals again. And if there's any preparation or information regarding that, I think we would like to know as preparation for ourselves as well” (sic) (CMED Student 5).

This was echoed by faculty members who expressed that it would be challenging and exhausting for students to return to practice sites in the future.

“So, they, you know, they may forget about that… because things that you don't practice, you know, they are more difficult to the get confidence in doing... so they're definitely not gonna be confident only from looking at videos” (sic) (CPH Faculty 1).

“In the future that they might need to spend more time in the hospital, you know, they might need to... to spend to get more effort so they learn as much as they can to compensate for the period, which had been lost” (sic) (CMED Faculty 4).

## 4. Discussion

This study highlights a set of challenges that QU Health students faced during their VI, which represented barriers to fulfilling the purpose of their training. Our results align with the findings of two systematic reviews that summarize the main weaknesses of virtual medical education in the era of COVID-19 (26, 36) regarding technical challenges, reduced student engagement, lack of essential skill development, loss of assessments, and the negative effect on the mental wellbeing of medical students.

A comparison of our results with those of a survey conducted at a university in KSA shows that medical students faced similar challenges with their VI; however, technical difficulties were more of a concern to those students than was seen in our study. Nevertheless, the majority of students in the KSA survey demanded the integration of the online expertise garnered during the pandemic into their practice and seemed to perceive some positive impacts of the pandemic on their training (29).

Moreover, QU Health students had to adapt to the new normal of medical education and were struggling with stress and uncertainty surrounding their future, a finding that compares well with data from other countries (1, 26, 27, 36–39). Such a finding is not surprising since this population of students is considered a vulnerable population, and medical students, globally, show higher rates of depression, suicidal ideation, and stigmatization around depression (27). Additionally, students were challenged by the lack of physical and mental support from peers and instructors and the lack of social engagement. This may decrease their motivation, increase their stress levels (39), and hinder learning (40).

Of note is that students from CPH and CMED were the most challenged among all QU Health students and suffered from the lack of clinical experience and having no real-life interactions with patients and healthcare professionals. This is similar to what was reported in studies conducted in different countries (1, 26, 30, 41). Not having the chance to play any role in dealing with the pandemic was also challenging to our students, and most of them struggled with feeling their worth in healthcare. This also coincides with what was reported by medical students across universities that applied the same containment approach during the pandemic (26, 28, 36, 39). In consistency with the available literature (1, 26, 30, 39–41), students also reported challenges with the ability to gain some important clinical and non-clinical skills and competencies and the availability of guidance and feedback. This was reflected in their perceived low confidence about achieving the goals of the VI and being in practice sites in the future. Based on the literature, all the above-mentioned factors may constitute barriers against the early formation of students' PI, which is a principal goal of medical education. This is based on PI being a social construct that highly depends on the nature of practice settings, on situational learning (5, 6, 11, 14, 42), and on acquiring the essential competencies for healthcare professionals (12, 17, 18). A lack of PI has a drastic negative impact on the students' perceived value of their profession as well as on the level of confidence that they need to become advocates of their professional opinions during their future medical practice (6, 42, 43).

Since physical interactions and patient contact are indispensable components of clinical teaching in different health professions programs, such extraordinary times demand innovations involving technology, and simulation-based teaching must be utilized. It would therefore be crucial to expand collaboration with telecommunication companies in order to provide and optimize the necessary interactive platforms for teaching and learning. The use of virtual conferences and social media should also be maintained as it was found to play a vital role in compensating for the loss of networking between students and other healthcare providers due to being away from training sites (36, 44). PI formation must be supported at all times (6), and it is crucial to avoid compromising it by creating a gap in the essential professional competencies and confidence needed to practice onsite. Given this, there is also a need for more studies that are focused on determining and measuring the short- and long-term effects of shifting to VI during the pandemic on the students' PI development.

Furthermore, problem-based learning and onsite training are the stages wherein students' professional competencies can be best measured (8, 28). Therefore, challenges imposed by the nature of VIs may additionally result in difficulty in measuring the competencies of health profession students, which is in accordance with what the faculty members and students involved in our study expressed about conducting assessments being one of the challenges faced during the VI. Our findings regarding challenges with assessments also resemble those from similar studies in the United Kingdom (UK), the Kingdom of Saudi Arabia (KSA), and Libya (29, 38, 39).

## 5. Study strengths and limitations

To enhance the rigor of our research study, we followed triangulation in different ways. First, we used multiple data sources to understand the challenges that the VI posed to students by conducting focus group discussions with students and individual interviews with clinical faculty members. Second, a different research team member who was not involved in the focus group discussion nor in conducting the individual interviews reviewed the transcribed data for validation. Third, independent data analysis was conducted by three members of the research team. In addition, the findings from some participants (preceptors/students) were confirmed by directing questions about them to the participants of other focus groups and interviews. However, this study has some limitations. We were not able to have the themes validated by the study participants, mainly due to time constraints. The study did not apply one specific validated framework, so focus group and interview guide questions did not specifically measure or assess the impact of shifting to VI on PI formation.

## 6. Conclusion

The impact of COVID-19 on health professions program training is only one part of the global impact it had on all aspects of normal daily activity and businesses. Meanwhile, remote teaching and Vis for students from different health professions programs, with all their challenges and drawbacks, have enabled medical education to continue despite the effects of the pandemic (39). Thus, our study results play an important role in identifying such challenges, among which are some inevitable barriers to virtual learning for medical students (26). Measures to help students adapt when faced with such challenges are essential given the uncertainty around the future persistence of the pandemic. It is necessary to provide students with mental health support, which can be facilitated by assigning mentors to help students adjust more rapidly to the COVID-19 crisis (45, 46). Our results also provide a better understanding of how such challenges and different experiences affect the development of students' PI. Hence, students, staff members, and policymakers alike should strive to minimize these barriers.

Finally, our study aligns with existing efforts to identify current issues and implement the necessary changes in a way that ensures that health professions education preserves its value, maintains its standards, and meets its ultimate goals.

## Data availability statement

The raw data supporting the conclusions of this article will be made available by the authors, without undue reservation.

## Ethics statement

The studies involving human participants were reviewed and approved by Qatar University Institutional Review Board (QUERG-CHS-2020-1). The patients/participants provided their written informed consent to participate in this study.

## Author contributions

HB, AE-A, AA-M, HA, and XD: conceptualization of the study, designing the interview guides, and critical review of the manuscript. RS: write-up of the manuscript. JM: data collection and write-up of the manuscript. GA-J: conceptualization of the study, designing the interview guides, data collection, data analyses, write-up of the manuscript, critical review of the manuscript, journal submission, and response to reviewer comments. All authors contributed to the article and approved the submitted version.
